# O072. An uncommon case of sinusal arrest in Cluster Headache

**DOI:** 10.1186/1129-2377-16-S1-A126

**Published:** 2015-09-28

**Authors:** Luciano De Biase, Caterina Santolamazza, Lidia D'Alonzo

**Affiliations:** Department of Clinical and Molecular Medicine, University Sapienza, St Andrew Hospital, Rome, Italy

It is well known how migraine attacks are strictly associated to abnormalities of cardiac rhythm. Aygun et al[1] have studied 30 patients during migraine attacks, observing abnormalities of rhythm (including sinus arrhythmia, atrial premature contraction and ventricular premature contraction), intervals greater than 0.2 seconds, corrected QT intervals greater than 0.44 seconds, T inversion and ST-segment abnormalities. However, the role of autonomic nervous system variations in migraine attack-related electrocardiographic changes remains uncertain. Moreover, in patients with migraine parasympathetic nervous system alterations were observed during normal daily activity in the headache-free period[2].

In July 2015, a 45-year-old man was referred to us for evaluation because of a recurrent migraine associated with bradycardia. He referred recurrent migraine attacks, frequently occurring at night and in the supine position. During the acute phases of the attacks he described bradycardia, and sometimes, thoracic oppression. Furthermore, our patient told us about a clinical history of panic attacks and renal colics with syncope.

He is currently taking topiramate and oxygen therapy at high doses (15 lt/min) during attacks.

In order to better understand the previous episodes of syncope and the phases of bradycardia occurrence during the pain attacks, we monitored his electrocardiogram for 24h.

During the 24h registration, he had three attacks of migraine preceded by warning signs, one of them at night time. Analyzing the ECG, we observed sinusal rhythm, medium CF 74 bpm (28-115 bpm), normal PR interval, rare supraventricular and ventricular premature contractions. In correspondence to the migraine attacks, we observed sinus bradycardia and sinus pauses with RR maximum interval of 5.5 seconds, sometimes with the occurrence of junctional rhythm, without loss of consciousness. Considering the results of this monitoring, we decided to implant a pace-maker.

This case demonstrates the importance of autonomic dysregulation in a patient with migraine, showing the need of further studies to better understand the phenomenon.

Written informed consent to publication was obtained from the patient(s).
